# Mediation of transactive memory capability in relationship of social media usage and job performance

**DOI:** 10.3389/fpsyt.2024.1361913

**Published:** 2024-05-29

**Authors:** Tao Feng, Ghulam Rasool Madni

**Affiliations:** ^1^ School of Management, Xi’an University of Architecture and Technology, Xi’an, Shaanxi, China; ^2^ Department of Economics, Division of Management and Administrative Science, University of Education, Lahore, Pakistan

**Keywords:** job performance, transactive memory capability, social media, structural equation modelling, China

## Abstract

This study explores the role of transactive memory capability as a mediator in the relationship between social media usage and job performance. Drawing on transactive memory theory, we hypothesized that individuals who use social media more intensively for task-oriented purposes and relationship building are more likely to develop transactive memory capability, which in turn enhances their job performance. A survey was conducted among 816 employees in China from various industries to collect data on their social media usage patterns, transactive memory capability, and job performance. Results from structural equation modeling indicate that social media usage has a positive impact on job performance. Additionally, transactive memory capability mediates the relationship between social media usage and job performance. This study has contribution in literature by demonstrating the beneficial effects of social media usage on the development of transactive memory capability and job performance. It is suggested that social media usage can be used a valuable tool for enhancing performance of employees. Employees should gain an understanding of how social media fosters the development of transactive memory capability to utilize it more effectively. These findings also suggest that the way individuals use social media can influence their ability to access and share knowledge within their social networks, ultimately impacting their job performance.

## 1 Introduction

Social media has a significant role in information acquisition due to its accessibility, diversity, and real-time nature because social media platforms provide updates of various topics, from news and current events to industry trends and personal interests but it is affecting memory of people (1). Sparrow et al. (2) introduced a novel perspective by investigating the effect of internet search on the “human memory system”. Rather than retaining actual knowledge, individuals remember the methods and location of acquiring information. Contemporarily, organizations are using social media tools to facilitate communications and exchanges of information (3). To put it differently, people now view memory as a wellspring of knowledge (4). Consequently, elucidating modern memory system that focus on recalling repositories rather than specific pieces of information proves to be an excellent approach. Social media can play a significant role in enhancing employee performance because social media platforms provide a space for employees to communicate and collaborate more effectively, regardless of their physical location. This can lead to improved teamwork and knowledge sharing (5). Active participation in social media can increase employee engagement by providing a platform for them to share ideas, receive feedback, and feel more connected to their organization. Social media can be used as a tool for informal learning, allowing employees to access a wide range of information and expertise that can contribute to their professional development. Employees who are active on social media can serve as brand advocates, helping to promote their organization’s values, products, and services to a wider audience.

Transactive memory is a concept that describes a cognitive system in which a group of individuals collectively store, organize, and retrieve information. It’s like a shared mental database where each person within a group specializes in certain types of information or knowledge, and they rely on one another to access this information when needed (5). Each individual in the group is responsible for specific types of information or expertise. They are seen as “experts” in their respective domains. Group members trust each other to store and retrieve the information they specialize (6). They depend on one another to fill in gaps in their own knowledge. Moreover, effective communication is essential for a transactive memory to work. Group members need to coordinate to access collective knowledge effectively (7). The emphasis is given on comprehending individuals who possess essential information and expertise rather than relying on memorization of facts (8). While existing studies concentrated on benefits of transactive memory within organizational contexts, the impact of social media for memory of individuals has gained much importance (9). Transactive memory allows teams to process information more efficiently by distributing the cognitive load across team members. Instead of each individual needing to know everything, they can rely on the expertise of others in the group. When a team member encounters a problem, they can quickly tap into the collective knowledge of the group through transactive memory, speeding up the problem-solving process. Knowing that others in the team have certain knowledge or expertise can reduce the cognitive load on individuals, freeing up mental resources for other tasks. Teams with strong transactive memory capabilities can make better decisions because they can access a wider range of expertise and perspectives. Developing transactive memory capability requires trust, effective communication, and a shared understanding of each team member’s expertise and responsibilities. Teams can improve their transactive memory by engaging in activities that promote knowledge sharing, such as regular team meetings, cross-training, and creating shared documentation or knowledge repositories.

Due to proliferation of different social networks, the widely adoption of smart phones, and increasing use of Internet, the effect of social media is rising. The utilization of social media has yielded a multitude of advantages, encompassing knowledge sharing activity, communications with a diverse array of individuals, and the formation of online groups (10). The wide-scale acceptance and social media have broadened people’s capacity to utilize this information. In this context, social media users harness their transactive memory capability to approach the plethora of available information on social networks (11). They utilize their transactive memory to sift through the data and avert information overload, allowing them to promptly gather high-quality information (12). However, all individuals do not possess the same level of transactive memory. “Factors like the degree of interpersonal connectivity, one’s aptitude for collecting and employing knowledge, and one’s willingness to do so all contribute to individual variances in transactive memory capability” (8). Consequently, “research on transactive memory at the organizational level has primarily focused on exploring the causal link between transactive memory and group productivity” (13). Only limited studies have examined the impact of transactive memory capability for job performance within organization.

The logic between social media usage and transactive memory capability lies in the way social media facilitate the sharing and storage of information among individuals and groups. Social media platforms provide a digital space where users can easily share, access, and store information, making it a valuable tool for enhancing transactive memory capability within teams or groups (10). It allows the users to share information, including documents, links, videos, and text posts. By sharing information, individuals contribute to the collective knowledge base of their team or group, enhancing transactive memory capability (11). It is also worth noting that social media make it easy to access information shared by others. This accessibility ensures that team members can quickly retrieve information when needed, improving the efficiency of knowledge retrieval and utilization (12). So social media facilitates collaboration and communication among team members. Through features such as group chats, discussion forums, and project management tools, team members can work together, share ideas, and coordinate tasks, thereby enhancing transactive memory capability (13). Moreover, social media platforms serve as external memory sources, allowing individuals to offload information from their personal memory to a shared platform. This externalization of memory helps in reducing cognitive load and improving transactive memory capability within the team (14). In addition, social media platforms provide tools for organizing and categorizing information, making it easier for team members to manage and access knowledge. This structured approach to knowledge management enhances transactive memory capability by facilitating the organization and retrieval of information (15). In essence, social media enhances transactive memory capability by enabling information sharing, improving knowledge accessibility, facilitating collaboration and communication, supporting memory externalization, and aiding in knowledge management. These factors develop a robust transactive memory capability within teams or groups, ultimately improving their performance.

China has one of the highest social media penetration rates in world, with over 989 million social media users in 2021. The popular social media in China include WeChat, Weibo, QQ, and Douyin (Chinese version of TikTok). WeChat is the most widely used platform, with over 1.2 billion users while Weibo has over 550 million active users. QQ, a messaging platform owned by Tencent, has over 600 million active users and Douyin has over 600 million users. Mobile devices are the primary means of accessing social media in China, with over 99% of users accessing social media through their smartphones. These statistics highlight the significant role that social media plays in China’s digital landscape and its impact on consumer behavior and communication.

The motivation behind this research is rooted in the growing influence of social media usage on various aspects of societies, including the workplace. With prevalence of social media platforms and their widespread adoption across industries, there is a need to understand how SOM impacts job performance. One key aspect of this relationship is the concept of transactive memory capability, which is crucial for effective teamwork and knowledge management, as it allows to leverage each other’s expertise and skills. The study investigates how SOM influences transactive memory capability and, in turn, how transactive memory capability affects job performance. By examining these relationships, the research seeks to provide insights into how organizations can leverage social media and enhance transactive memory capability to improve overall job performance.

## 2 Literature review and hypotheses

Social media platforms facilitate information sharing and collaboration among individuals and groups. Users often share knowledge, insights, and resources, fostering the development of transactive memory (14). Each individual becomes a source of information within their network. Social media can increase job performance because professionals use social media for networking, staying informed about industry trends, learning new skills, and accessing resources (15). Additionally, it makes efficient communications and collaborations within teams, leading to improved task management and project outcomes. Transactive memory system within a team or organization can boost job performance (16). When individuals rely on knowledge and expertise of their colleagues, they can make more informed decisions, solve problems more efficiently, and allocate resources effectively. This collective knowledge-sharing increases overall performance of the firms (17). Social media acts as a catalyst for the development of transactive memory by promoting knowledge-sharing and collaborative learning (18). As social media users engage with their networks, they build and rely on their transactive memory systems (19). Users can tap into the collective knowledge of their network when seeking information. It allows them to efficiently access information and make more informed decision, thereby increasing the utility of social media (20). Ultimately, the combination of SOM and the development of transactive memory capability within a team or organization can lead to improved job performance (5). Teams that effectively use their collective knowledge tend to perform better because they can access expertise and resources more efficiently (21). The strength of these relations can vary depending on individuals and organizational factors. Factors like the extent of SOM, knowledge sharing culture, and the effectiveness of transactive memory within the organization play important role (22). Social media promotes knowledge-sharing and collaborative learning, which contributes to developing transactive memory capability (10). transactive memory capability, in turn, enhances the benefits of social media and leads to improved job performance. This dynamic relationship emphasizes the importance of effectively integrating these elements in a professional context to optimize knowledge-sharing and overall performance (14).

A transactive memory capability is a collective knowledge sharing and storage mechanism that a group or organization employs to enhance its overall cognitive capabilities. It is a concept introduced by psychologists (4). In a transactive memory system, individuals within a group or organization specialize in certain types of information or expertise. They are considered “experts” in their respective domains. Group members trust each other to store and retrieve information they specialize in (23). They depend on one another to access knowledge and fill in gaps in their own understanding. Effective communication is vital for a transactive memory system to function optimally (24). Group members need to share information and coordinate with each other to access the collective knowledge efficiently (25). People within the group typically possess complementary skills and knowledge. This means that each individual’s expertise complements that of others, making the group collectively knowledgeable and efficient (26). The group can retrieve information more efficiently than individuals working in isolation. When someone needs specific information, they can ask the group member responsible for that knowledge, reducing the efforts needed to find information (12). When one member is uncertain, they can cross-check with others, which is especially valuable in decision-making and problem-solving situations (27). When a member leaves the group or new information becomes available, the system can adjust by redistributing knowledge and responsibilities (28). Transactive memory systems are often observed in various settings, such as teams, families, and organizations (5). Transactive memory is vital for efficient knowledge management and decision-making, especially in complex and knowledge-intensive environment (29, 30).

There are two overarching approaches leveraging transactive memory capability. The first approach involves remembering the specific locations of essential information on social media. This method entails committing to memory where relevant data can be found. By doing so, individuals establish a foundation for seamless access to the required knowledge, ensuring it is always readily available (31). The second method centers on utilizing “transactive memory formed through interactions with individuals possessing the necessary information. In this approach, transactive memories are built through interpersonal engagements, and they serve as reservoirs of knowledge acquired from these interactions” (32). However, the advent of social media platforms has eliminated these constraints, allowing for the enhancement of transactive memory capability through interactions unrestricted by time or location (5, 33). Through social media, individuals can establish connections with a multitude of other people, offering numerous advantages for utilizing it as an external memory. This heightened connectivity enables active knowledge and information sharing (34). Furthermore, positioning oneself favorably within these networks opens up opportunities for better information access and knowledge absorption (35, 36). Moreover, using external memory reduces the burden of having to recall all information, leading to a reduction in memory overload.

### 2.1 Social media and transactive memory capability

Online platforms create boundaries for establishing satisfied relation and trust compared to real-life situation. Developing a substantial presence on social media can help overcome these challenges, offering advantages over offline environments in terms of trust-building (35). Additionally, forming relation with individuals in an online setting, where communication is unrestricted by time and location, can lead to greater achievements. Social media provides a highly beneficial environment for connecting with others in the network and expressing thoughts online. The mobile environment has further enhanced this connectivity, allowing social media users to communicate anytime and anywhere, fostering closer connections than community media (36).

The presence on social media helps to establish knowledge-based trust necessary for improving an individual’s transactive memory capability and enables more effective communications and interaction among members, eliminating communication barriers and creating an environment necessary for building relations among distant groups (24). Community members also exhibit a sense of social cohesions, enhancing activities that contribute to knowledge. Furthermore, members’ behaviors become more influential, with clear and honest communication fostering knowledge sharing activity. Social media, in this regard, offers a highly advantageous environment for connecting and creating opportunities for online expression of thoughts and ideas (37). Additionally, the ubiquity of mobile devices in today’s world has created an environment for social media users to engage in communication anytime and anywhere, fostering deeper connections among users compared to community-based media.

Social media communications make it easier to assess others’ knowledge, ultimately generating a transactive memory. “Strong relationships and frequent social interactions are associated with improvements in transactive memory capability” (38). Increasing the quantity and quality of relationships enhances trust in peers, making it easier to determine if others have specialized knowledge. Additionally, social interactions, whether direct or indirect, increase the specific knowledge. Research on transactive memory capability indicates that “interactions related to one’s job responsibilities significantly influence the formation of transactive memory within a team, broadening opportunities for understanding and using knowledge. Through new social online interactions, social media users establish stronger relationships, actively share knowledge and information, and engage in personal, cultural, and emotional exchanges, thus increasing their transactive memory capability by fostering trust and attribution” (39).

In this context, robust relationship and frequent social interaction is directly linked in transactive memory capability. Expanding both quantitative and qualitative dimensions of relationships increases trust and simplifies the evaluation of whether others possess specialized knowledge in specific domains (40–44). Furthermore, these interactions not only enable the direct exchange of specific knowledge but also provide opportunities for indirect connections with others. In essence, social interactions, whether direct or indirect, heighten the probability of encountering others’ specialized knowledge (5). Existing literature underscores the significance of interactions, particularly those tied to one’s job responsibilities, in shaping transactive memory (20, 39, 45). Repetitive interactions also play a critical role in forming transactive memory capability within teams, broadening the scope for knowledge comprehension and utilization (33). “Through new social online interactions, social media users establish stronger connections with others, actively share knowledge and information, and participate in personal, cultural, and emotional exchanges grounded in these intimate relationships” (46).

*H1: Social media usage has positive impact on transactive memory capability of individuals.*

### 2.2 Job performance and transactive memory capability

Transactive memory empowers individuals to effectively harness new information and knowledge. Individuals with high transactive memory proficiency can efficiently acquire and utilize information, giving them a competitive edge in processing knowledge more swiftly than others. Various components of transactive memory enhance team level job competency (13). Notably, it is discovered that transactive memory capability exerts a greater effect on team job competencies compared to other factors (9). Additionally, implementing a transactive memory capability with aid of information fosters knowledge sharing (10). Firms with well-established transactive memory capability benefit from successful knowledge-sharing activity (47). Collaboratively established transactive memory systems within organizations enhance the informational capacity of those involved in building the system (4, 48, 49). Transactive memory substantially enhance individuals’ capacity to assimilate new knowledge (25). The stronger the transactive memory capability, the more favorable the communication that accompanies its formations, ultimately bolstering individuals’ operational capabilities (50). Individuals with robust transactive memory capability have a heightened potential to acquire various types of knowledge as they serve as central figures within networks (35). The five most critical dimensions of social media have diverse effects on transactive memory capability (51).

*H2: Job performance of individuals has positive relationship with transactive memory capability.*

### 2.3 Social media usage and job performance

Social media platforms offer a vast repository of information, insights, and expertise (52, 53). Users can follow industry leaders, access educational content, and stay updated on the latest trends. This knowledge acquisition enhances one’s understanding and skills, which can directly contribute to job performance (14). Social media enables individuals to connect with professionals, colleagues, and experts. This connection create opportunity for mentorship, collaboration, and the exchange of ideas and experiences (25). Building a robust professional network can be invaluable for career growth and improved job performance. Many social media platforms offer educational content, webinars, and online courses (32). Social media platforms provide communication and collaboration tools that facilitate problem-solving and teamwork (45). Teams can use these platforms to collaborate on projects, share documents, and hold discussions (48). Efficient collaboration contributes to better job performance, particularly in team-based environments (38). Social media provides insights into customer behavior, market trends, and competitor activities. This information is crucial for making informed business decisions and optimizing strategies, which can positively impact job performance, especially in marketing and sales roles (44).

Employees’ utilization of social media aid firms in realizing their objectives by fostering network resources referred to as “Social Media Capital” (54). Through the social media usage, employees gain access to a greater pool of resources, such as information and knowledge, which, in turn, promotes exchange of information (55). Employees who have access to abundant resource via social media tend to experience reduced workplace stress and exhibit a higher propensity to engage in behaviors that extend beyond their defined job roles. One such behavior is actively contributing to and assisting their colleagues in their responsibilities, a practice that enhances interpersonal cooperation and commitment to the workplace (56). Additionally, the presence of social media, from a technical standpoint, enhances visibility and makes it more convenient for employees to connect and collaborate with their peers, thereby enhancing operational efficiency (32). Conversely, it also aids in circumventing redundant work and the repetitive execution of tasks, thereby boosting productivity and refining job performance (57). For instance, employees can utilize and adapt readily available information to formulate appropriate solutions when relevant situations arise, eliminating the need to duplicate efforts (32).

*H3:* Social media usage has positive relationship with job performance of individuals.

## 3 Theoretical foundations

The theoretical foundations draw upon several key concepts and theories from the fields of organizational behavior, psychology, and communication. Transactive Memory Theory, proposed by Wegner, explains how “groups collectively encode, store, and retrieve information” (31). According to this theory, “groups develop a shared memory system where each member specializes in specific types of information, creating a transactive memory system that enhances group performance” (58).

The aspect of social media usage and job performance draws on theories related to the effect of technologies on workplace behavior and performance. Social media usage in the working place can affect productivity, collaboration, and information sharing. Theories such as media richness theory and social influence theory may be relevant here, explaining how different communication channels (including social media) can impact performance outcomes. The study also draws on various theories of job performance, such as “goal-setting theory, expectancy theory, or social exchange theory” (32). These theories help to explain how individual behaviors, motivations, and interactions within some work environment influence job performance outcomes. According to Gibson’s model, “team thinking requires interaction, remembering, and communicating among members, as well as negotiating, interpreting, and evaluating” (46). The Information Processing Theory suggests that leaders help the teams to coordinate their time by organizing information, learning about each other’s progress, understanding deadlines, and reducing uncertainty. This collaboration helps team members connect mentally, identify the right person for a task, share memories, build trust, and work together better, leading to a better team memory system and improved use of team knowledge.

**Figure 1:**
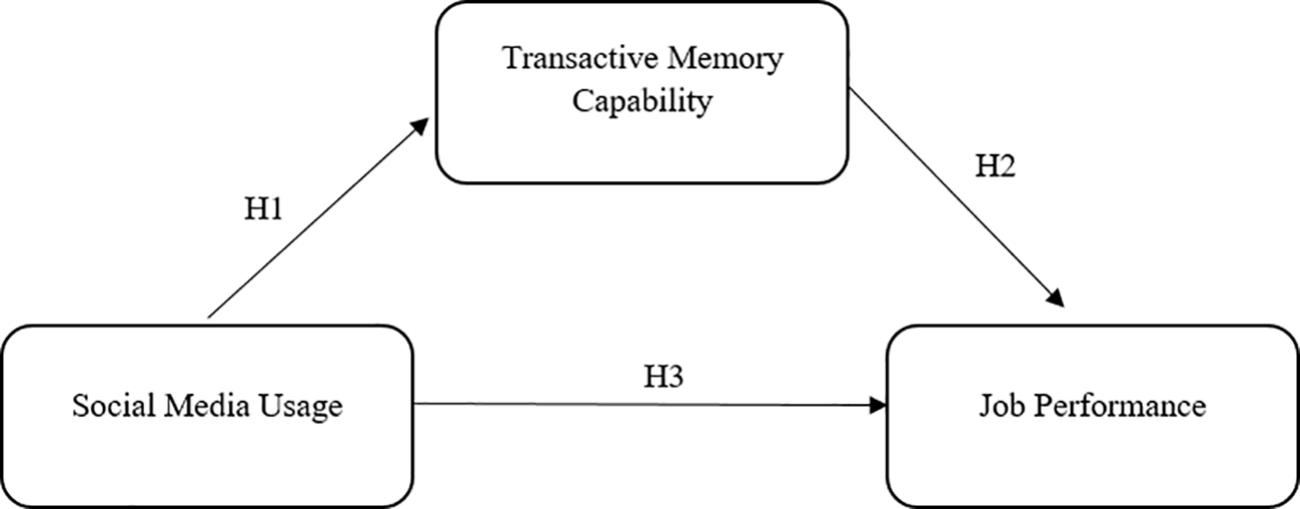
Research Model.

The theoretical framework of the study integrates these various theories and concepts to propose a comprehensive model. It likely posits that social media usage affects job performance indirectly through its impact on transactive memory capability, suggesting that effective use of social media can enhance collective memory systems within organizations, leading to improved performance outcomes. Overall, the theoretical foundations of this research article combine insights from transactive memory theory, theories of social media usage and technology use, job performance theories, and mediation analysis to provide a comprehensive analysis of how social media usage influences job performance through the mediation of transactive memory capability. The research model is shown in **Figure 1**.

The study’s conceptual framework, is based on existing literature, which also identifies a gap in the literature. In this model, social media is considered an independent variable, while transactive memory capability is seen as a mediator variable, and job performance is the dependent variable.

## 4 Materials and methods

Data collection was conducted using online resources. Convenience sampling was employed, aiming for a broader participation of population of the study. Individuals meeting the study criteria were personally contacted through personal and professional networks, particularly those working in knowledge intensive firms such as IT, R&D, consultancy, and healthcare. The respondents were selected if they are using any social media platform. The survey was disseminated via social media platforms like, LinkedIn, Instagram and Facebook. People who met the criteria for this study, from personal and professional networks were personally approached. This means that people who work in more than one team simultaneously in knowledge-intensive organizations were approached. The link was sent via an online link, the participants received a reminder every week to complete the survey in order to obtain a sufficient number of respondents. To address language barriers, the survey was available in both English and Chinese languages. A written consent is gained from participants and questionnaires were distributed and collected from employees of those companies established for over 6 months. Data was collected in China during June-August 2023. In total, 1120 questionnaires were sent and 816 of them were selected as suitable. Participants were excluded from the sample if they are below the age of 18 years or they are unemployed. Depending on the specific focus of the study, the response of those participants is not considered if they do not use social media or if they use it excessively, beyond the scope of the study. Moreover, incomplete filled survey forms were also excluded. The descriptive analysis is given in **Table 1**.

**Table 1:** Descriptive analysis (N=816).

Categories	Items	Frequency	%age
Gender	Male	412	50.49
	Female	404	49.51
Education	High School	39	4.78
	Bachelor	452	55.39
	Master	301	36.89
	PhD	24	2.94
Marital Status	Married	501	61.40
	Single	315	38.60
Age	18–29 Years	301	36.89
	30–39 Years	299	36.64
	40–50 Years	196	24.02
	More than 50 Years	20	2.45
Residence	Urban Area	446	54.66
	Rural Area	370	45.34

### 4.1 Estimated results

This study utilized scales from previous research, which were measured on 7 point Likert-type scale ranging from “strongly disagree” to “strongly agree.” Appendix 1 includes the questionnaire, describing the details of all constructs and number of items. Survey questions were designed using existing scales from the literature to enhance validity. Social media usage was adapted from studies by Carlson et al. (59) and Moqbel et al. (60) The measure of TMC was adapted from Lewis’s research. The JP scale was adapted from Huang and Liu’s study. Structural equation modeling is employed to evaluate the data. The findings of the internal reliability are reported in **Table 2**.

**Table 2:** Reliability and validity test.

Variable	Symbol	Factor Loading	CR	CA	AVE	KMO	Barlett’s test		
							χ^2^	df	Sig
Task Oriented Usage of Social Media Usage (TMC)	“TMC 1	0.79	0.84	0.83	0.85	0.80	746	5	0.00
	TMC 2	0.72							
	TMC 3	0.81							
	TMC 4	0.77							
Relationship Building Usage of Social Media (RSM)	RSM 1	0.85	0.78	0.83	0.82	436	3	0.00	0.85
	RSM 2	0.89							
	RSM 3	0.82							
	RSM 4	0.76							
Intensity of Social Media Usage (ISM)	ISM 1	0.88	0.92	0.85	0.83	0.79	1539	5	0.00
	ISM 2	0.81							
	ISM 3	0.79							
	ISM 4	0.85							
Transactive Memory Capability (TMC)	TMC 1	0.66	0.82	0.81	0.84	0.77	637	4	0.01
	TMC 2	0.69							
	TMC 3	0.72							
	TMC 4	0.85							
	TMC 5	0.86							
Job Performance (JP)	JP 1	0.82	0.87	0.82	0.86	0.77	613	4	0.01
	JP 2	0.81							
	JP 3	0.73							
	JP 4	0.79							
	JP 5”	0.77							

“CR, Construct reliability; CA, Cronbach’s alpha; KMO, Kaiser–Meyer–Olkin; AVE, Average variance extracted.”

The value of CA is more than 0.7 showing internal consistency of the variables. “The convergent validity is measured through CR, Barlett’s test, and KMO values. The empirical findings reveal that CR values are more than standard value of 0.8, KMO values higher than acceptable value of 0.7, and significance level is 0.000, qualify for Bartlett’s test. The discriminant validity is determined through Pearson’s correlation coefficient and the AVE square root of each variable” (52). The findings of discriminant validity are shown in **Table 3**.

**Table 3:** Discriminant validity.

	TMC	JP	SMU	TMC	RSM
**TMC**	0.88				
**JP**	0.45	0.85			
**SMU**	0.62	0.63	0.86		
**TMC**	-0.51	-0.42	-0.59	0.85	
**RSM**	0.60	0.40	0.55	-0.49	0.78

“All values are significant at 1% while diagonal value is square root of AVE”.

The “correlation coefficients are smaller than square root of AVE depicting that internal correlations between the observed variables are higher than external correlations, so discriminatory validity exists” (41).

### 4.2 Structural equation modeling

SEM is used to investigate the effect of social media usage on job performance and transactive memory capability. SEM is a “statistical technique used to analyze the relationships between observed variables and latent constructs. It allows researchers to test complex theoretical models that include multiple variables and pathways” (59). In SEM, researchers specify a model that hypothesizes relation between latent variables and observed variables (which are directly measured). The model is then tested using data to see how well it fits the observed data. SEM can be used for various purposes, such as testing theories, exploring causal relationships, and validating measurement instruments. The analysis in SEM involves “estimating the parameters of the model (e.g., regression coefficients, factor loadings) and assessing the overall fit of the model to the data. Goodness-of-fit indices, such as the chi-square test, Comparative Fit Index (CFI), and Root Mean Square Error of Approximation (RMSEA), are used to evaluate the model fit. SEM is a powerful tool for testing and refining theories, as it allows researchers to examine the relationships between multiple variables simultaneously and to assess the overall validity of their theoretical models” (60). The findings of SEM are presented in **Table 4**.

**Table 4:** Structural parameters.

Hypotheses	Path	t-values	Result
SOM→TMC	0.416*	6.475	Yes
TMC→JP	0.387*	7.185	Yes
SOM→JP	0.484*	9.275	Yes

* shows significance at 1%.

The findings highlight that all paths are significantly positive so all hypotheses are supported. The empirical analysis reveal that SOM positively affects the transactive memory capability, confirming hypothesis 1. The second hypothesis is also confirmed that TMC enhances the JP while empirical analysis also support that SOM positively affects the JP.

Confirmatory Factor Analysis is a widely used statistical method for assessing the validity of a measurement instrument. It allows researchers to test how well a proposed model fits the data. Various goodness of fit statistics are used to evaluate the extent to which the model’s expected relationships among variables match the actual relationships observed in the data. CFA enables researchers to model error variance and assess the adequacy of the factor structure. To determine the suitability of the constructs, CFA was conducted (**Table 5**).

**Table 5:** Confirmatory factor analysis.

Confirmatory Factor Analysis
x^2^/df = 2.976*RMR = 0.059 NFI = 0.901IFI = 0.913 CFI = 0.910RFI = 0.921 GFI = 0.912RMSEA = 0.073

The CFA confirmed that the model was a good fit based on the fit indices. The “(x2/df) ratio was below 5, and the RMR score was 0.063, while the RMSEA score was 0.079, both below the acceptable threshold of 0.08. Additionally, the GFI, CFI, RFI, NFI, and IFI values were all greater than the recommended values of 0.90” [].

The mediation analysis, as depicted in **Table 6**, reveals that TMC serves as a partial mediator in the relation between SOM and JP. The outcomes indicate that TMC partially mediates the association between SOM and JP. Based on these findings, we can conclude that SOM significantly and indirectly impacts JP through the mediation of TMC. In essence, the utilization of social media yields an indirect influence on job performance. This conclusion is substantiated by the confidence intervals generated via bootstrapping.

**Table 6:** Mediation efect.

Effect of SOM on TMC	Effect of TMC on JP	Direct	Indirect	Confidence Interval		P-value	Result
				L Bound	U Bound		
0.33	0.38	0.43**	0.14**	0.074	0.151	0.001	Partial

** show significance at 5%.

## 5 Discussion

This study investigates the effect of SOM on TMC and how TMC, in turn, influences an individual’s job performance. The findings demonstrate that the model exhibits satisfactory reliability and validity. Moreover, the results reveal that all path coefficients are statistically significant. To begin, the outcomes of this study provide substantiation for H1, which postulates a positive relationship between SOM and TMC. This implies that social media platforms offer a range of functionalities, including communications and coordination system, repositories of knowledge, and mechanisms for discovering and accessing information, within the realm of social media (31). This endorsement of SOM supports the notion that it enhances the effectiveness of TMC among individuals within an organization. Social media usage can positively affect TMC in several ways. Social media usage platforms provide a convenient and efficient means for individuals to share information, knowledge, and resources (58). This sharing fosters a culture of collaboration and information exchange, contributing to the development of transactive memory capability. Because interactions on social media often involve discussions, debates, and knowledge-sharing. Engaging in such discussions exposes individuals to different perspectives and insights, which can enrich their own understanding and contribute to the collective knowledge base, enhancing transactive memory capability (59). Collaborative efforts on social media platforms, such as group chats or project-specific channels, can foster a sense of teamwork and interconnectedness among colleagues (60). This strengthens the bonds among team members and enhances transactive memory capability as they learn to rely on each other for information (53). It can be summarized as that SOM positively affects TMC by facilitating information sharing, knowledge exchange, and collaboration. It encourages the development of interconnected cognitive systems within teams or communities, enabling individuals to rely on one another’s expertise and memory, ultimately enhancing their collective transactive memory capability (61). If organization employs social media for discussions, knowledge creation, and information exchange, while simultaneously expanding their knowledge repository, the influence of SOM on TMC will be amplified (34, 46, 62). Social media platforms provide a convenient and accessible way for team members to share information, knowledge, and expertise. By using social media, team members can contribute to a shared pool of knowledge, enhancing the team’s transactive memory capability.

Social media encourages individuals to externalize their knowledge by posting, commenting, and sharing content. This process of externalization helps to make individual knowledge more visible and accessible to other team members, improving the team’s overall transactive memory. It also allows teams to connect with a broader network of individuals both within and outside the organization. By expanding their network, teams can access a wider range of expertise and knowledge, further enhancing their transactive memory capability. The study (51) examined the impact of social media use on knowledge sharing within teams. The researchers found that teams that used social media to share knowledge had higher levels of transactive memory capability compared to teams that did not use social media. Another research (47) investigated the relationship between social media use and team performance in virtual teams. The researchers found that social media use positively influenced transactive memory capability, which in turn led to higher team performance. This study examined the impact of social media use on knowledge sharing and team performance. The researchers found that social media use significantly enhanced knowledge sharing and improved team performance.

The findings of the study substantiate Hypothesis 2, which postulated a positive correlation TMC and JP. transactive memory capability emerges as a crucial element that exerts a positive influence on an individual’s JP. Previous research has consistently demonstrated the impact of transactive memory capability on JP (5, 25, 35, 48, 50). Transactive memory capability has proven to be a significant factor in enhancing the JP. Transactive memory capability allows individuals to tap into the collective knowledge and expertise of a group or team. When faced with a task, team members can quickly retrieve information and solution from their colleagues’ memory, making problem-solving more efficient (55). Instead of trying to remember everything individually, team members can rely on others who specialize in specific fields. This reduces the mental burden and allows to focus on their core responsibilities, leading to better performance. With transactive memory capability, teams can make quicker and more informed decision (61). Team members can access relevant information and expertise within the group, enabling them to make well-informed choices without having to conduct extensive research. As experienced team members share their knowledge with newer colleagues, it accelerates the learning for newcomers, helping them adapt to their roles more swiftly (51). Team members with diverse knowledge and expertise can collaborate effectively, combining their ideas and insights to develop innovative solutions. Transactive memory capability suggests that teams with strong TMC are more efficient and effective because they can access a broader range of knowledge and expertise. According to TMC theory, teams with strong transactive memory can process information more efficiently. Instead of each individual needing to know everything, they can rely on the expertise of others in the group. This reduces duplication of effort and speeds up decision-making processes. In addition, TMC theory suggests that transactive memory allows teams to allocate cognitive resources more effectively. Individuals can focus on their areas of expertise, knowing that they can rely on others for different types of knowledge. This leads to better resource utilization and higher job performance. TMC theory also emphasizes the importance of knowledge integration. Teams with strong transactive memory can combine diverse knowledge and perspectives, leading to more innovative solutions and better performance. Earlier research (12, 38, 46) found that teams with higher levels of transactive memory performed better on a decision-making task than teams with lower levels of transactive memory. The researchers concluded that transactive memory enhances team performance by improving information processing and decision-making. The study (32) examined the relationship between transactive memory and team performance in software development teams. The researchers found that teams with stronger transactive memory capabilities were more productive and produced higher quality software than teams with weaker transactive memory. A study (44) investigated the relationship between transactive memory and team performance in a simulated task environment. The researchers found that teams with higher levels of transactive memory performed better on a complex decision-making task than teams with lower levels of transactive memory.

The study outcomes validate Hypothesis 3, affirming a positive correlation between SOM and JP. Social media serves as a tool for preventing time wastage and repetitive tasks in the workplace, ultimately enhancing productivity (33, 55–57). Social media usage can improve JP in different ways, depending on how it is integrated into one’s professional life and used effectively. While social media is a vast source of information and educational content. An employee can follow industry news, access webinars, and stay updated on the latest trends. This continuous learning can contribute to expertise and JP (63). In addition, social media allows to monitor customer feedback, industry trends, and competitors’ activities. This market intelligence can inform strategies, helping to make informed decisions and stay competitive. Moreover, social media platforms often offer collaboration tools and these tools facilitate teamwork and effective communication with colleagues and team members. Drawing from previous research, it can be deduced that transactive memory capability acts as a mediator that augments individual JP via the conduit of social media (64). It enables them to harness the collective intelligence of their network, retrieve information efficiently, and make informed decisions. These mechanisms, in turn, lead to enhanced JP.

The study reveals that relationship between SOM and JP is mediated by TMC and this finding could highlight the importance of transactive memory in a digital age and the role of social media in facilitating it. It is found that individuals who are highly skilled in using social media platforms demonstrate significantly better transactive memory capability, which in turn leads to improved job performance. This finding could underscore the importance of digital literacy and expertise in the workplace. The study uncovers that the diversity of social media platforms used by individuals positively correlates with their transactive memory capability, which in turn influences job performance. This finding could suggest that a diverse social media usage pattern may contribute to better performance in the workplace. The study suggests specific interventions or strategies that organizations can implement to enhance TMC through SOM, leading to improved job performance. These could include training programs, policy changes, or technological enhancements. These findings could provide valuable insights for researchers, practitioners, and organizations interested in understanding the complex interplay between social media, transactive memory, and job performance, encouraging further exploration and the generation of new knowledge in this area.

### 5.1 Implications

#### 5.1.1 Theoretical implications

The study could contribute to the understanding of how transactive memory capability, which refers to the ability of a group to encode, store, and retrieve knowledge collectively, is influenced by social media usage. This study extends the literature confirming the transactive memory theory, media richness theory and social influence theory, expectancy theory, or social exchange theory. It could shed light on how social media usage can facilitate the development and utilization of transactive memory within teams or organizations. The research could offer insights into how social media usage can be integrated into organizational learning models. It could provide a framework for organizations to leverage social media tools to enhance their transactive memory capabilities and, ultimately, their job performance. The findings could have implications for human resource management practices, particularly in terms of team composition and training. Understanding how social media and transactive memory interact could help HR professionals build more effective teams and develop training programs that leverage social media for knowledge sharing and collaboration.

#### 5.1.2 Practical implications

Organizations can use platforms of social media to improve team collaboration by facilitating sharing of knowledge and communication. By enhancing transactive memory capabilities through social media, teams can work more effectively together, leading to improved job performance. Social media may be used as a tool for knowledge management, allowing organizations to capture, store, and share knowledge more efficiently. By leveraging social media for knowledge management, organizations can improve their transactive memory capabilities and enhance job performance. With the rise of remote work, social media can provide a platform for remote teams to collaborate and share knowledge. By using social media platforms to enhance transactive memory capabilities, organizations can support remote work arrangements and maintain productivity. Organizations can use social media for training and development purposes, helping employees build their transactive memory capabilities. By providing training through social media platforms, organizations can improve job performance and enhance overall organizational effectiveness. Social media can facilitate organizational learning by providing a platform for employees to share insights, lessons learned, and best practices. By using social media to enhance transactive memory capabilities, organizations can promote a culture of continuous learning and improvement.

### 5.2 Limitations and future research

The findings of the study may be limited in their generalization due to limited sample size, which could limit the statistical power of the analysis. Future research could use larger samples to increase the reliability of the findings. The study’s measures of social media usage, transactive memory capability, and JP may have limitations in terms of validity and reliability. Future research may use more robust measurement tools to improve the results in more precision.

Future research may be extended by conducting longitudinal studies to examine how social media usage and transactive memory capability evolve over time and their impact on job performance. Moreover, conducting cross-cultural studies to explore how social media usage and transactive memory capability vary across different cultural contexts and their impact on job performance.

## 6 Conclusions

With the increasing popularity of social media, an expanding number of organizations are incorporating it into their operations to enhance communications and collaborations. However, research on the social media usage in the workplace remains limited. In response, we have constructed a model to investigate the impact of social media usage and the underlying mechanism through which it generates value in a work context, by combining the concepts of media synchrony and transactive memory capability. As demonstrated, social media contributes to the development of transactive memory capability, exerting a significant influence on JP. Our findings quantify the advantages of social media for organizations, thereby encouraging employees to embrace its use within the workplace. Organizations can enhance employee JP by adhering to the guidelines outlined in this study.

This study is particularly focused on examining the impact of social media usage on JP of employees within Chinese organizations. The outcomes of the study have crucial implications. Firstly, by considering the mediating role of transactive memory capability, this research bridges a gap in understanding the effect of social media usage on JP. Secondly, it contributes to the theoretical comprehension of dynamics between social media usage, transactive memory capability, and JP. This study augments literature by revealing the positive consequences of employee social media usage on the development of transactive memory capability and JP. Additionally, it offers further insights into how social media usage can enhance transactive memory capability and employee JP.

This study holds several practical implications. It underscores that social media serves as a legitimate communications tools for fostering workplace connectivity. Organizations must also comprehend how social media usage fosters the development of transactive memory capability, enabling more effective utilization. Therefore, management should educate employees on maximizing the utility of social media to enhance their opportunities for professional growth. A vital contribution of this study is the inclusion of transactive memory capability as a mediator within the model. Our research reveals that employees possess transactive memory capability, which, in turn, impacts JP through their use of social media.

Despite the valuable insights this study provides, it also has its limitations. Many questions remain unanswered, paving the way for future research endeavors. Firstly, our focus on subjective indicators may not entirely capture the objective reality. Secondly, the cross-sectional nature of data does not account for the gradual development of transactive memory capability, which evolves over time through ongoing interactions and collaboration. Future research should explore how social media contributes to the gradual development of transactive memory capability. Lastly, the generalizability of our findings may be questioned, given the specific focus on China, which possesses distinct cultural values. Future research should aim to apply these findings to diverse cultural contexts, thereby reducing such uncertainties.

## Data availability statement

The raw data supporting the conclusions of this article will be made available by the authors, without undue reservation.

## Ethics statement

The studies involving humans were approved by Ethics Committee of University of Education. The studies were conducted in accordance with the local legislation and institutional requirements. The participants provided their written informed consent to participate in this study. Written informed consent was obtained from the individual(s), and minor(s)’ legal guardian/next of kin, for the publication of any potentially identifiable images or data included in this article.

## Author contributions

TF: Data curation, Formal analysis, Methodology, Writing – original draft. GM: Conceptualization, Writing – original draft.
